# Effects of antibiotic and disinfectant exposure on the mouse gut microbiome and immune function

**DOI:** 10.1128/spectrum.00611-24

**Published:** 2024-09-18

**Authors:** Wen-Bo Xu, Yun-Fan Wang, Si-Yu Meng, Xiao-Tong Zhang, Yi-Rong Wang, Zhao-Ying Liu

**Affiliations:** 1 College of Veterinary Medicine, Hunan Agricultural University, Changsha, China; 2 Hunan Engineering Technology Research Center of Veterinary Drugs, Hunan Agricultural University, Changsha, China; Hong Kong University of Science and Technology, Hong Kong, Hong Kong

**Keywords:** disinfectant, antibiotic, *NLRC4*, gut healthy, immunity response

## Abstract

This study explores the effects of disinfectant and antibiotic exposure on gut health, focusing on gut microbiota balance and gut immune function. Our analysis indicates that disinfectants increase the proportion of Gram-positive bacteria, particularly increasing *Staphylococcus* levels, while antibiotics increase the proportion of Gram-negative bacteria, especially *Bacteroides* levels. These changes disrupt microbial harmony and affect the gut microbiome’s functional capacity. Additionally, our research reveals that both disinfectants and antibiotics reduce colon length and cause mucosal damage. A significant finding is the downregulation of *NLRC4*, a key immune system regulator in the gut, accompanied by changes in immune factor expression. This interaction between chemical exposure and immune system dysfunction increases susceptibility to inflammatory bowel disease and other gut conditions. Given the importance of disinfectants in disease prevention, this study advocates for a balanced approach to their use, aiming to protect public health while minimizing adverse effects on the gut microbiome and immune function.

**IMPORTANCE:**

Disinfectants are extensively employed across various sectors, such as the food sector. Disinfectants are widely used in various sectors, including the food processing industry, animal husbandry, households, and pharmaceuticals. Their extensive application risks environmental contamination, impacting water and soil quality. However, the effect of disinfectant exposure on the gut microbiome and the immune function of animals remains a significant, unresolved issue with profound public health implications. This highlights the need for increased scrutiny and more regulated use of disinfectants to mitigate unintended consequences on gut health and maintain immune system integrity.

## INTRODUCTION

The gut functions not only as a digestive organ but also plays a crucial role in maintaining overall health and immune system balance (1). The symbiotic relationship between the host and the gut’s extensive microbial ecosystem is vital for the immune system’s development and functionality (2, 3). These microorganisms are essential for food digestion, nutrient synthesis, and immune defense modulation against pathogens (4). Disruptions in gut microbiota equilibrium are closely linked to various diseases, including inflammatory bowel diseases (IBDs) like Crohn’s disease and ulcerative colitis, as well as obesity and cardiovascular diseases (5–7). Moreover, the significance of the gut extends beyond nutrient absorption and immunological defense to the profound impact of its complex microbial ecosystem—known as the gut microbiota—on overall health (8–10). Maintaining a healthy balance within the gut microbiota is crucial for promoting wellbeing and preventing the imbalances that can lead to diverse health challenges (11).

Current research indicates that antibiotics, while beneficial, can also be harmful, with increasing evidence linking their overuse to various disorders related to alterations in gut microbiota (12–14). Studies show that prolonged antibiotic use can reduced beneficial gut microbiota and increase the proportion of harmful organisms (15). Additionally, antibiotics facilitate the horizontal transfer of antibiotic resistance genes, enhancing bacterial resistance (16, 17). The imbalances caused by antibiotics in gut microbiota extend beyond gastrointestinal issues to broader health concerns, including immune system effectiveness, obesity, and inflammatory conditions (12, 18–20). Bactericidal antibiotics may also disrupt the metabolic activities of myeloid cells, diminishing their ability to eliminate bacterial pathogens (21, 22), thus increasing vulnerability to secondary infections.

Recently, disinfectant use has surged in animal husbandry, the food processing industry, homes, and hospitals (23, 24). This widespread application has led to environmental contamination, affecting water and soil quality and promoting bacterial resistance (25, 26). Such developments pose a risk of cross-infection and exacerbate antibiotic resistance, highlighting the growing threat that disinfectants pose to human health (27–29). Despite these concerns, research on the impact of disinfectants on the gut microbiome remains unclear.

To address this issue, a model was constructed to determine whether disinfectants affect the gut microbiome and immune function. Benzalkonium chloride (BAC), a widely used quaternary ammonium disinfectant, has broad-spectrum biocidal activity against bacteria, algae, fungi, and viruses (30). BAC was chosen to study the effect of disinfectant exposure on microbial antibiotic resistance (31). This model aims to understand how disinfectants disrupt the gut ecosystem and affect immune regulation. Oxytetracycline (OTC), a broad-spectrum tetracycline antibiotic, was selected as the antibiotic group (32, 33). Widely used in medicine, veterinary medicine, and agriculture, OTC serves as a model for assessing the impact of antibiotics on gut health (34–37). This study assesses the differences in the impact of disinfectants versus antibiotics on the gut, offering insights into their distinct mechanisms of action and potential health implications.

## MATERIALS AND METHODS

### Animals and experimental design

All experimental procedures were approved by the Hunan Experimental Animal Center and Hunan Drug Safety Evaluation and Research Center (Changsha, China) and were conducted in accordance with the Guidelines for the Care and Use of Laboratory Animals of the National Institutes of Health (Batch Number: 2023–022). Female ICR mice weighing 18–22 g obtained from Hunan SJA Laboratory Animal Co., Ltd. were acclimated to the experimental environment for at least 7 days with *ad libitum* access to food and water. After 7 days of acclimation, the mice of each breed were randomized into two groups administered with the following for 8 weeks: Control (control and sterile saline), OTC (oxytetracycline 0.1 mg/mL) (38), and BAC (benzalkonium chloride 0.005%) (39). Solutions were delivered via drinking water and refreshed twice weekly (12). After 8 weeks, animals were euthanized by carbon dioxide inhalation. Samples were collected and stored immediately at −80°C for further analysis.

### Determination of organ bacterial burdens

After 8 weeks of treatment, the animals were euthanized, and their organs were weighed. Gastrointestinal tissues were extracted aseptically, longitudinally opened into thin sections, and submerged in 10 mL of sterile phosphate-buffered saline (PBS). The tissues were vigorously shaken to remove the contents. The washed tissues were then added to 5 mL of Hank’s Balanced Salt solution (HBSS) supplemented with 5% fetal bovine serum (FBS) and 25 mM 4-(2-hydroxyethyl)−1-piperazineethanesulfonic acid (HEPES), shaken vigorously, and the supernatant is stored in fresh tubes (designated as the “mucus layer’). This step is repeated to produce 10 mL of the “mucus layer,” which is then centrifuged at 4,000 rpm for 4 minutes. The pellet is resuspended in 1 mL of sterile PBS. The tissues are finally washed twice in sterile PBS (each time shaken in 5 mL), and the remaining tissue is homogenized in 1 mL of sterile PBS before plating (designated as “deep tissue layer”). Plate culture was performed on MacConkey (Solarbio, China) for Gram-negative bacteria and Columbia nalidixic acid (CNA) selective agar (Hopebio, China) for Gram-positive bacteria to determine the bacterial load (40). Colony counts were performed after incubation at 37°C for 24–48 hours according to the method described by Drummond (12, 41).

### Preparation of genomic DNA from murine fecal samples

Microbiome analysis was performed on fecal samples collected using sterilized forceps from mice housed in sterilized empty cages. Samples were stored at −80℃ until DNA extraction. Total genomic DNA was extracted using the cetyltrimethylammonium bromide (CTAB) method, and its concentration and purity were monitored on 1% agarose gels (42). The DNA was diluted to 1 ng/µL using sterile water based on its concentration.

### 16S amplicon sequencing and analysis

The preparation of 16S rRNA sequencing libraries was carried out accordance to Novogene (China) protocols. The V3–V4 hypervariable region of the bacterial 16S rRNA gene was universal bacterial primers (515F: GTGCCAGCMGCCGCGGTAA, 806R : GGACTACHVGGGTWTCTAAT) (designed by Novogene). Sequencing libraries were generated using the Illumina TruSeq DNA PCR-Free Library Preparation Kit (Illumina, USA), and index codes were added. Library quality was assessed using the Qubit@ 2.0 Fluorometer (Thermo Scientific, USA) and Agilent Bioanalyzer 2100 system. The library was sequenced on an Illumina NovaSeq platform to generate 250-bp paired-end reads.

### Bioinformatics analyses

Paired-end reads were merged using fast length adjustment of short reads (FLASH) and assigned them to each sample based on unique barcodes. Sequences were analyzed using quantitative insights into microbial ecology (QIIME) and in-house Perl scripts to assess α-diversity and β-diversity. Quality-filtered reads were processed using pick_de_novo_otus.py to generate operational taxonomic units (OTUs) with a 97% similarity threshold. Representative sequences were selected for each OTU, and taxonomic information was annotated using the RDP classifier (43). We computed α-diversity metrics by rarifying the OTU table and calculating Chao1, observed species, and Shannon indices. Rarefaction curves were generated based on these metrics.

For β-diversity, weighted UniFrac was used as a phylogenetic measure, with principal coordinate analysis (PCoA) and unweighted pair group method with arithmetic mean (UPGMA) clustering. PCoA transformed the distance matrix into orthogonal axes, visualizing the maximum variation with the first principal coordinate and the second maximum with the second principal coordinate. UPGMA clustering used average linkage to interpret the distance matrix (44).

To further analyze microbial diversity differences between samples, we conducted significance tests using statistical analysis methods such as *t*-test and linear discriminant analysis effect size (LEfSe), which were used to analyze microbial diversity differences, with LEfSe indicating significant differences by an LDA score >2 and *P*-value < 0.05.

### Histopathology

Colon samples were fixed in 4% paraformaldehyde, embedded in paraffin, and sectioned and stained with haematoxylin and eosin (H&E) for histological examination to determine the severity of inflammation and the extent of mucosal and crypt damage (45, 46).

### RT-qPCR analysis of gene expression

Total RNA was isolated using TRIzol reagent (Accurate Biotechnology, China) and reverse-transcribed into cDNA using the ABScript Neo RT Master Mix for qPCR with gDNA Remover (ABclonal, China). RT-qPCR was performed using a LightCycler 480 Instrument II (Roche Diagnostics) with BlasTaq 2 × qPCR MasterMix (abm, Canada) to measure the mRNA expression, with *GAPDH* as a reference gene. Relative mRNA expression was normalized to *GAPDH* and determined using the 2−ΔΔCt method (47). All primer sequences are listed in Table 1.

**TABLE 1:** Primer sequence (designed by this study)

Gene	Primer F (5’−3’)	Primer R (5’−3’)	Product (bp）
*NLRC4*	TGTGTGAGCAGTGACGGATG	CAGTGCTGCATCCGGTAAGA	136
*AIM_2_*	ACCCGCAGTGACAATGACTT	CAGCACCGTGACAACAAGTG	118
*Caspase-1*	ACTGACTGGGACCCTCAAGT	GCAAGACGTGTACGAGTGGT	111
*Caspase-11*	ACTGAGGTATGGGGCTAAC	CTTTCACCACCACATCGT	145
*IL-18*	TCAGACAACTTTGGCCGACT	GGTGGATCCATTTCCACTTTGA	122
*IL-1β*	GCAACTGTTCCTGAACTCAACT	ATCTTTTGGGGTCCGTCAACT	138
*TNF-α*	CCCTCACACTCAGATCATCTTCT	GCTACGACGTGGGCTACAG	133
*ASC*	CTCGCTGACATCATCCTCGC	GCGTTTTGTTGCCGTAGCAG	172
*NLRP3*	AGCTGGGATTAGACAACTGC	CATTGTTGCCCAGGTTCAGC	192
*GAPDH*	TGGAGATTGTTGCCATCAACG	TGCCGTGAGTGGAGTCATAC	113

### Enzyme-linked immunosorbent assay

The quantification of interleukin-1β (*IL-1β*), interleukin-18 (*IL-18*), and apoptosis-associated speck-like protein containing CARD (*ASC*) levels in mouse colon tissue was performed using an enzyme-linked immunosorbent assay (ELISA) kit supplied by AiFang (Changsha, China). This analysis adhered strictly to the protocol provided by the manufacturer.

### Statistical analysis

Phenotypic clinical evaluation and colonic key immune factor content data were analyzed using SPSS statistical software with the *t*-test. Statistical significance was based on a *P*-value < 0.05 (*P < 0.05; **P < 0.01).

## RESULTS

### Changes in the gut microbiota caused by disinfectants and antibiotics

To determine the impact of antibiotics and disinfectants on gut microbiota stability, we studied the effects of long-term exposure to OTC and BAC on the gut microbiota of mice. The results indicated significant alterations in the microbial communities within the gut tissues of mice subjected to prolonged exposure to these substances.

**Fig 1:**
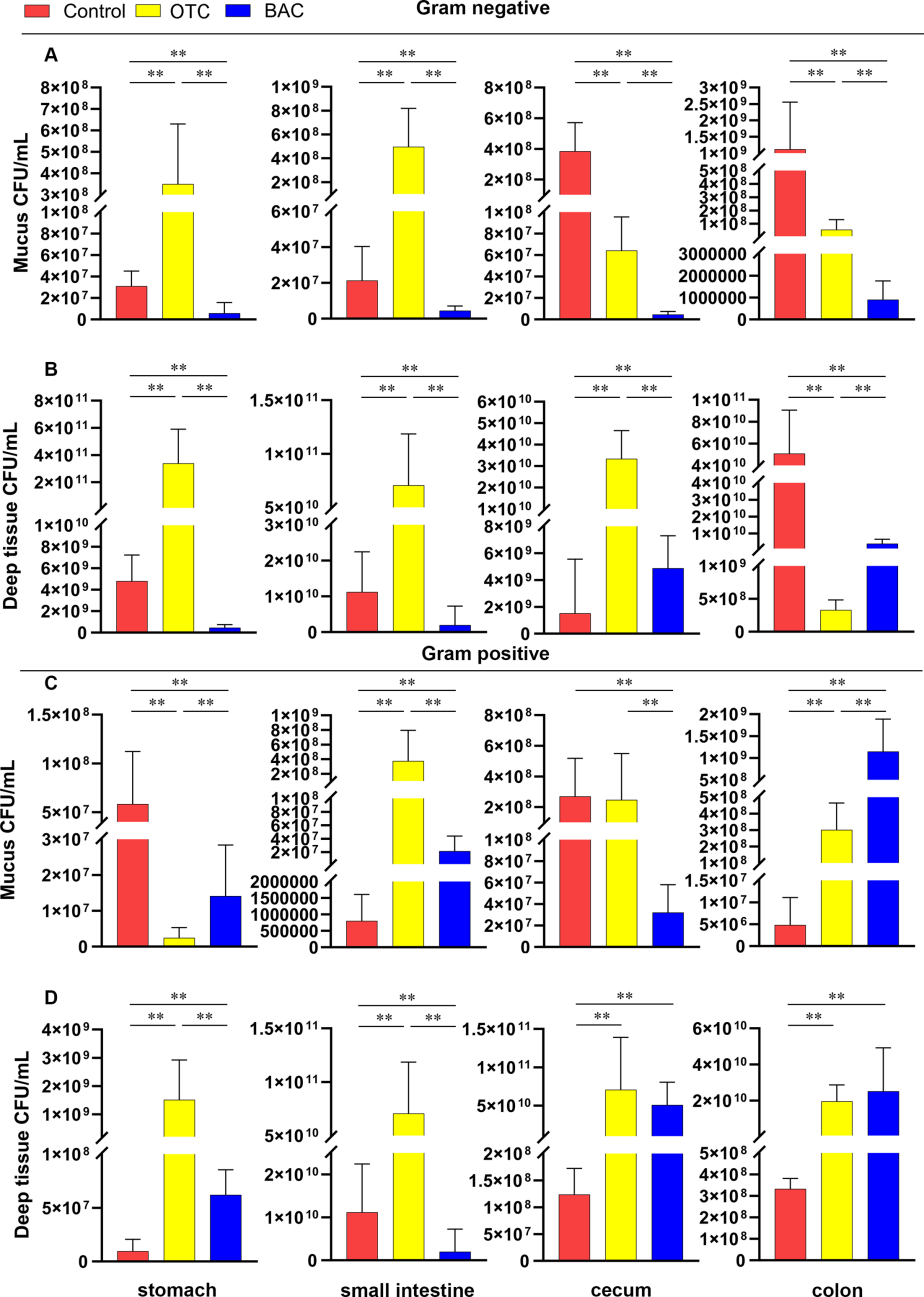
OTC and BAC induce dysbiosis in the mouse gut microbiota. (**A**) Gram-negative bacterial load in the gut mucosal layer of the control group (*n* = 10), OTC group (*n* = 10), and BAC group (*n* = 10) mice. (**B**) Gram-negative bacterial load in the deep tissue layer of the gut tract in mice. (**C**) Gram-positive bacterial load in the gut mucosal layer in mice. (**D**) Gram-positive bacterial load in the deep tissue layer of the gut tract in mice. Data were collected from three independent experiments and analyzed by *t*-test. *P < 0.05; **P < 0.01.

Mice exposed to OTC exhibited an increase in the proportion of Gram-negative bacteria in the mucosal layer of the stomach and sections of the small intestine, with a decrease in bacterial content in the cecum and colon (Fig. 1A). Conversely, there was a decrease in Gram-positive bacteria in the stomach, while an increase was noted in parts of the small intestine and colon, and no significant change was observed in the cecum (Fig. 1C). In the deeper tissue layers, an increase in Gram-negative bacteria was observed in the stomach, parts of the small intestine, and cecum, while a decrease was noted in the colon (Fig. 1B). Gram-positive bacteria showed an increase in proportion across the stomach, small intestine, cecum, and colon (Fig. 1D).

On the other hand, mice exposed to BAC showed a decrease in Gram-negative bacteria in the mucosal layer of the stomach, small intestine, cecum, and colon (Fig. 1A). There was also a decrease in Gram-positive bacteria in the stomach, small intestine, and cecum, with an increase in the colon (Fig. 1C). In the deeper tissue layers, a decrease in Gram-negative bacteria was observed in the stomach, small intestine, and colon, while an increase was found in the cecum (Fig. 1B). Gram-positive bacteria increased in the stomach, cecum, and colon, but decreased in the small intestine (Fig. 1D).

These findings illustrate significant shifts in the gut microbial communities under the long-term influence of OTC and BAC. Notably, the effects varied between mice exposed to OTC and those exposed to BAC, underscoring the differential impact these substances have on gut microbiota.

### 16S amplicon sequencing analysis of the impact of disinfectants and antibiotics on the gut microbiome

**Fig 2:**
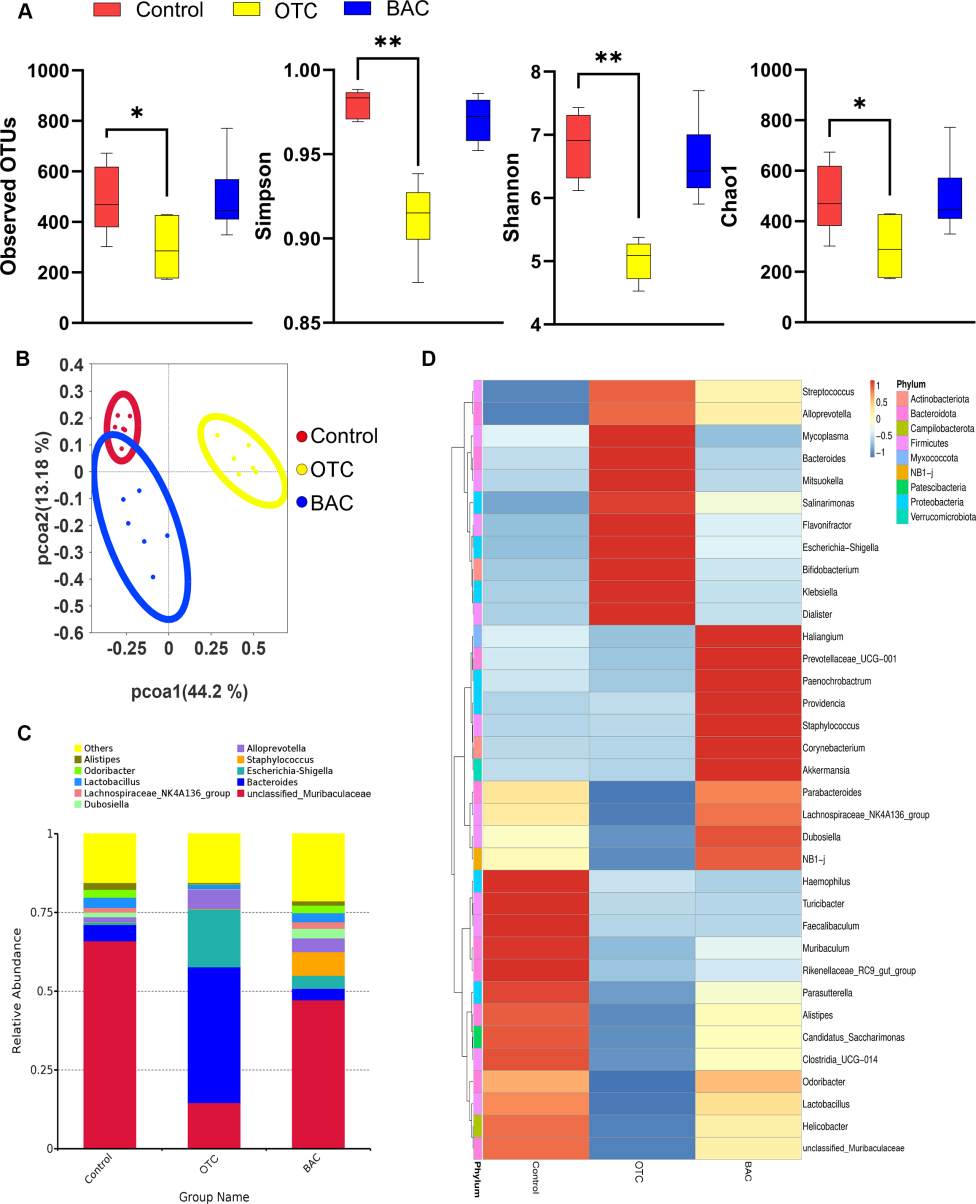
Changes in the gut colon microbiota composition of mice after exposure to OTC and BAC. (**A**) Comparison of observed gut microbiota OTUs and Shannon, Simpson, and Chao1 indices between the control group, OTC group, and BAC group (*n* = 6 samples/group). (**B**) Principal coordinate analysis (PCoA) plot of microbiota composition spectra among control, OTC, and BAC groups (*n* = 6 samples/group). (**C**) Genus-level relative abundance bar charts for control, OTC, and BAC groups (*n* = 6 samples/group). (**D**) Genus-level species abundance cluster heatmap for control, OTC, and BAC groups (*n* = 6 samples/group).

We used richness and Shannon index as indicators of species diversity within the sample to measure the α-diversity and used the *t*-test method for statistical analysis. The α-diversity between the BAC group and the control group showed no significant difference, whereas the OTC group has significantly lower α-diversity when compared to the control group (Fig. 2A).

Using the Bray–Curtis distance algorithm and analyzed using the Tukey statistical method, significant differences in β-diversity were observed between the control group and both the OTC and BAC groups, with notable differences also between the OTC and BAC groups (Table 2). This indicates that both OTC and BAC induce distinct alterations in the gut microbiota (Fig. 2B).

**TABLE 2:** Diversity calculation

Group 1	Group 2	Sample size	*P*-value
BAC	Control	12	0.00995
Control	OTC	12	0.004975
BAC	OTC	12	0.014925

Microbial composition analysis revealed diversified communities of different bacterial genera in both the BAC and OTC groups, as shown by relative abundance bar graphs and species abundance cluster heatmaps (Fig. 2C and D). In the OTC group, the proportion of *Bacteroides* significantly increased, while *unclassified*_*Muribaculaceae* (48) decreased compared to the control group. In the BAC group, *Staphylococcus* significantly increased. Additionally, *Bacteroides* and *Escherichia-Shigella* (belonging to Bacteroidota and Proteobacteria, respectively) significantly increased in the OTC group. In the BAC group, *Staphylococcus* and *Dubosiella* (both belonging to *Firmicutes*) significantly increased (Fig. 2C).

Each column of the heatmap was normalized and analyzed, revealing three bacterial categories. Bacteria in the control group were mainly specialized gut bacteria, closely associated with host health, and mostly anerobic or microaerophilic. Probiotics like *Lactobacillus* play a crucial role in maintaining gut health and balance (49). The OTC group primarily contained common gut symbionts involved in food digestion and nutrient absorption, such as *Bacteroides* and *Bifidobacterium*, which are mostly anerobic or facultatively anerobic (50). The BAC group included bacteria from various environments, including opportunistic pathogens like *Staphylococcus* and *Corynebacterium*, which can cause infections under certain conditions (51, 52)(Fig. 2D).

**Fig 3:**
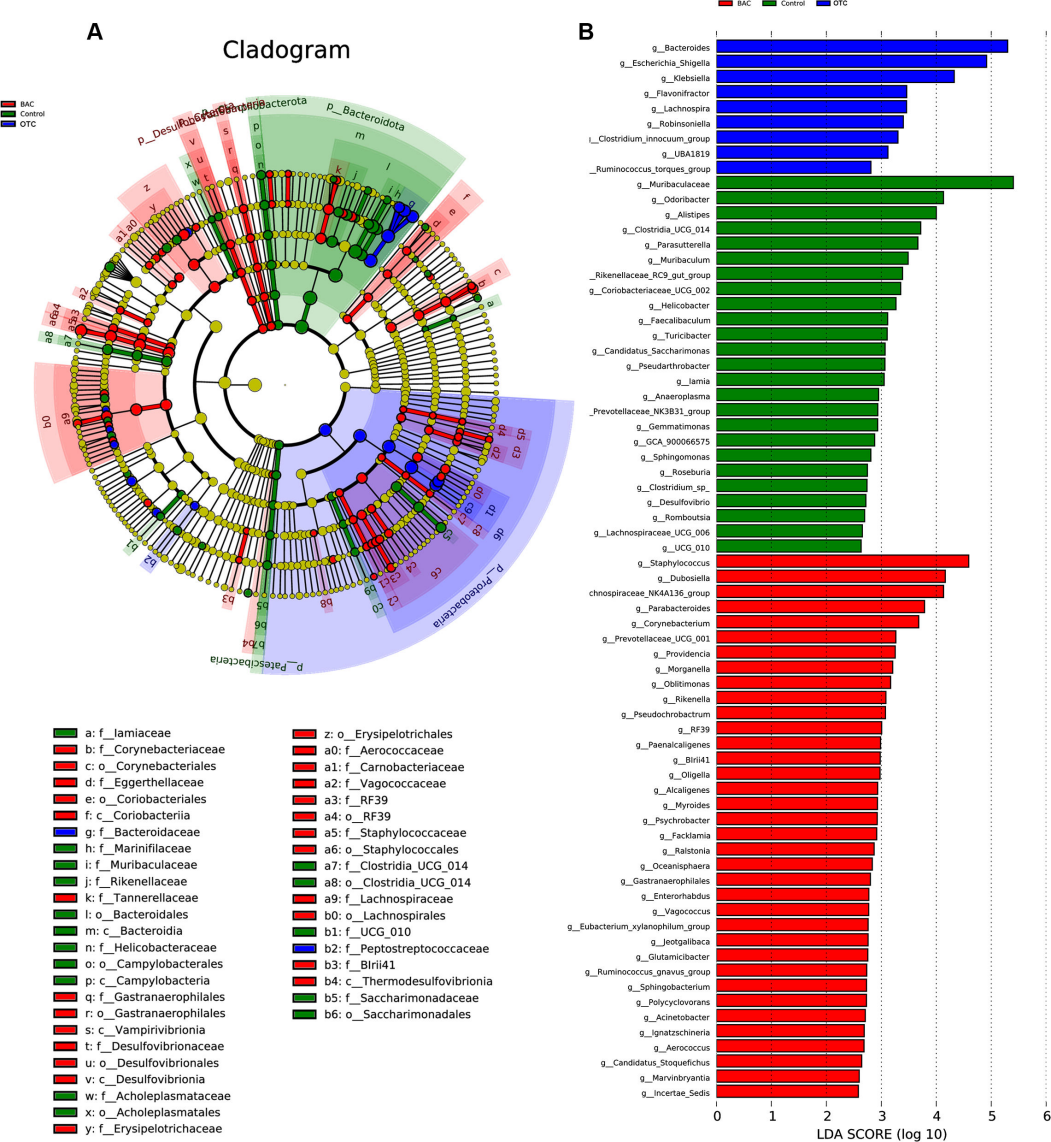
Differences in microbial abundance between control, OTC, and BAC groups (*n* = 6 samples/group). (**A**) Cladogram. (**B**) LDA distribution. Linear discriminant analysis effect size (LEfSe) was used to analyze differences in the microbial abundance across the three groups.

To identify specific bacterial genera unique to different groups, we employed linear discriminant analysis effect size (LEFSe) (Fig. 3A). Our data indicate that in the OTC group, there was a significant enrichment of the genera *Bacteroides* and *Escherichia-Shigella*, which are typical Gram-negative bacteria from the phyla Bacteroidetes and Proteobacteria, respectively. In the BAC group, typical Gram-positive bacterial genera such as *Staphylococcus* and *Lachnospiraceae_NK4A136_group* were significantly enriched, consistent with previous findings (Fig. 3B).

### Damage to gut immune function by disinfectants and antibiotics

#### H&E staining results

**Fig 4:**
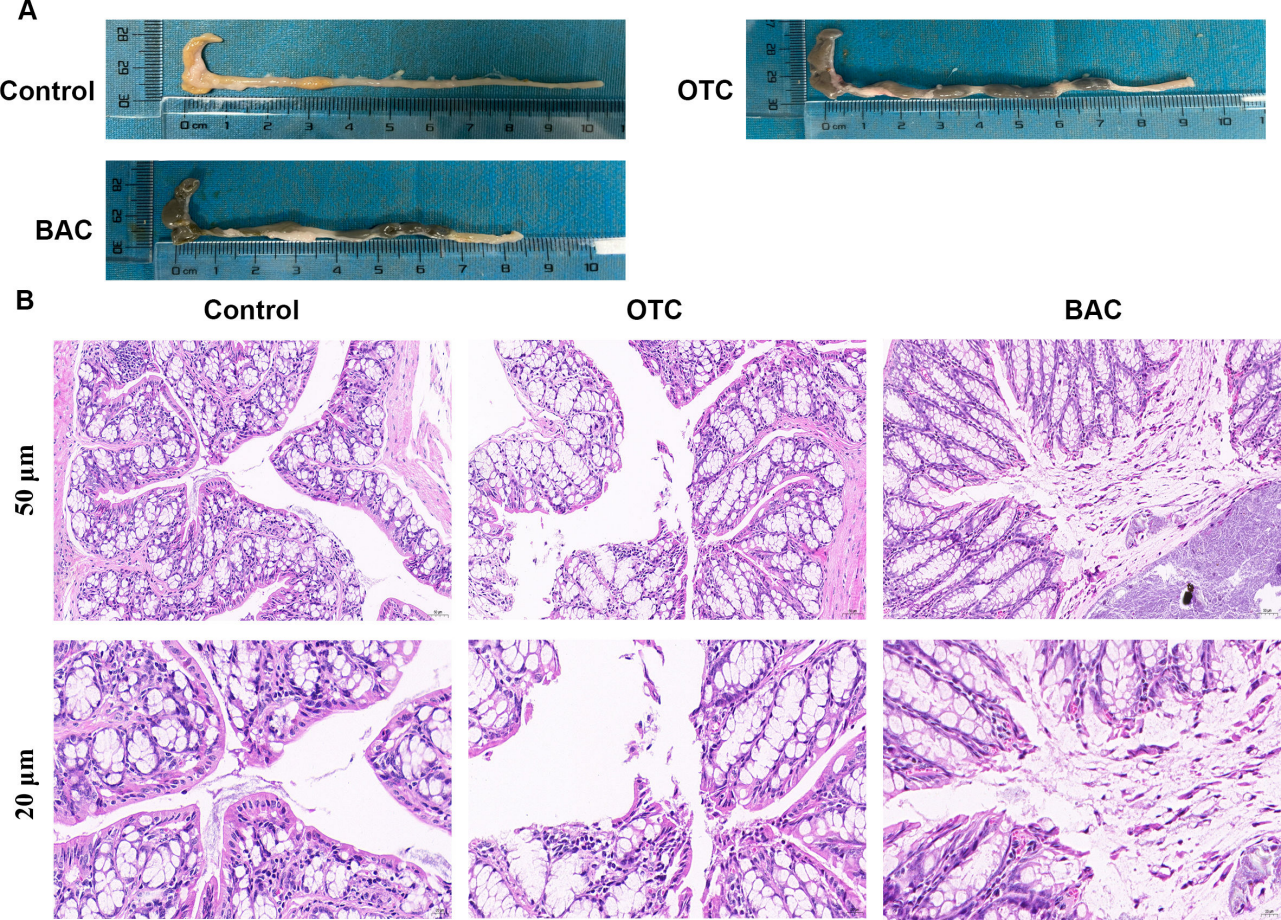
Histopathology of the mouse colon tissue after treatment with OTC and BAC. (**A**) Colon length in control, OTC, and BAC groups. (**B**) H&E staining of the colon tissue in control, OTC, and BAC groups.

Both OTC and BAC pre-exposed mice exhibited a reduction in colon length, with the reduction being more pronounced in the BAC pre-exposed group (Fig. 4A). H&E staining revealed that the colonic mucosal epithelium in the control group and OTC pre-exposed group was intact and clearly visible, with orderly arranged epithelial cells. In contrast, the BAC pre-exposed group showed signs of mild degenerative necrosis and epithelial shedding in the colonic mucosal epithelium (Fig. 4B).

#### RT-qPCR analysis of gene expression and ELISA results

**Fig 5:**
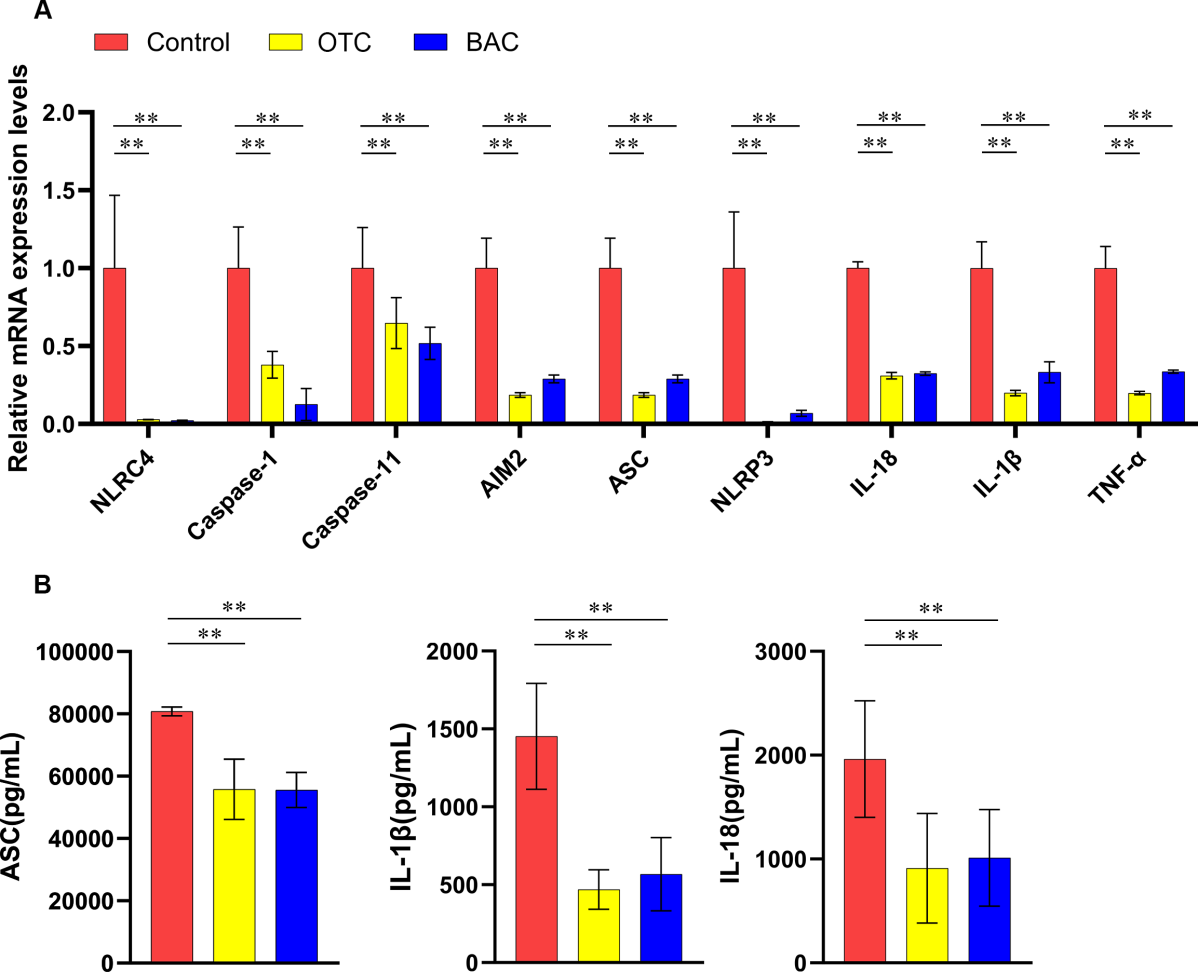
mRNA and protein detection in the mouse colon after exposure to OTC and BAC. (**A**) qPCR detection of mRNA expression levels of NLRC4, caspase-1, caspase-11, AIM2, NLRP3, IL-18, IL-1β, and TNF-α in the colon of mice from control, OTC, and BAC groups. (**B**) ELISA detection of ASC, IL-1β, and IL-18 protein expression levels in the colon of mice from control, OTC, and BAC groups. Data were collected from three independent experiments and analyzed by *t*-test. *P < 0.05; **P < 0.01.

RT-qPCR analysis revealed a significant decrease in the expression of inflammasome components, including *NLRC4*, *caspase-1*, *caspase-11*, *AIM2*, *ASC,* and *NLRP3*, as well as proinflammatory cytokines *IL-18*, *IL-1β*, and *TNF-α* (Fig. 5A). ELISA results corroborated these findings, showing significantly reduced levels of *ASC*, *IL-18*, and *IL-1β* in protein extracts from the colon (Fig. 5B).

These results strongly suggest that both BAC and OTC can impact gut immunity by modulating the production of gut cytokines. The mechanism through which BAC exerts its effects may involve the disruption of cell membranes. While this characteristic is essential for its antimicrobial action, it can also cause damage to host cells, including those in the gut mucosa. This cellular damage could impair the gut barrier function and immune response, highlighting the need for careful consideration when using disinfectants like BAC, particularly in contexts where they might impart gut microbiome and overall gut health.

## DISCUSSION

The impact of disinfectant exposure on the gut microbiome and immune function of animals remains a significant, unresolved question with profound implications for public health. Few studies have examined the long-term effects of disinfectants on animals. Our findings show that disinfectant exposure disrupts the gut microbiome, leading to a significant increase in Gram-negative bacteria. In contrast, antibiotic exposure results in a higher presence of Gram-positive bacteria. These results demonstrate that disinfectants and antibiotics affect the gut microbial through different mechanisms.

BAC is known to effectively destroy lipopolysaccharides, a key component of the outer membrane of Gram-negative bacteria (39). This makes Gram-negative bacteria more susceptible to BAC, particularly in regions where BAC concentrations are higher. As a result, there is a reduction in Gram-negative bacteria in various parts of the gut. The decrease in Gram-negative bacteria typically leads to reduced competition for Gram-positive bacteria (53). Consequently, Gram-positive bacteria can proliferate in certain gut sections. This shift in bacterial populations disrupts the overall gut environment, highlighting the complex and profound effects of disinfectant exposure on gut microbiome stability and immune function. Meanwhile, OTC can increase both Gram-negative and Gram-positive bacteria in the gut. This suggests that OTC may disrupt the microbial balance by inhibiting the growth of specific bacteria and promoting the growth of resistant strains. Additionally, the dynamic interactions among gut microbiota, such as competition and cooperation, along with the host’s immune response and mucosal secretions, further influence the relative abundance and distribution of different bacterial groups (54). These combined factors explain the complex effects of OTC on the changes in Gram-negative and Gram-positive bacteria levels in various gut regions.

OTC can effectively kill tetracycline-resistant *Escherichia coli* and inhibit *Actinobacillus pleuropneumoniae*, a pathogen responsible for swine pneumonia (55, 56). Additionally, BAC has demonstrated significant bactericidal effects on *Listeria monocytogenes* (57). Numerous studies have documented the impact of antibiotics on intestinal microbes (58–60), and our findings corroborate these studies. Specifically, OTC administration elevates the presence of Gram-negative bacteria in the gut microbiome, such as *Bacteroides* and *Escherichia-Shigella*, aligning with previous reports (61). Interestingly, our study reveals that disinfectants exert an effect on the gut microbiota, which contrasts with that of antibiotics. BAC exposure results in a heightened abundance of Gram-positive bacteria, such as *Staphylococcus*, in the gut. Infections with *Staphylococcus* species can lead to a range of adverse outcomes in animals, including increased inflammatory responses, compromised gut barrier integrity, and immune system disruption, resulting in difficult-to-manage conditions (62–64). This finding introduces a novel perspective on how disinfectants interact with the gut ecosystem, significantly contrasting with the well-documented influence of antibiotics. Throughout the experiment, we monitored the mice’s weight and found no significant changes. However, the feces of the mice in the BAC group were looser and softer than those in the control group. The precise mechanisms underlying these effects remain elusive, necessitating further investigation to elucidate the detailed pathways through which disinfectants distinctly influence the gut microbiota.

*NLRC4* is a critical protein within the NOD-like receptor (NLR) family, playing a vital role in regulating the cellular immune response (65). It recognizes specific pathogen-associated molecular patterns (PAMPs) and damage-associated molecular patterns (DAMPs) from host cells, thereby activating the immune system (66–70). *NLRC4* is notably triggered by the bacterial type III secretion system (T3SS), stimulating the production of pro-inflammatory cytokine and inflammatory responses to counteract pathogens (71–73). Additionally, alterations in the *NLRC4* gene are linked to autoimmune diseases, activating the inflammasome and leading to gut inflammation (74–76). A decrease in *NLRC4* expression can heighten infection risks, disrupt gut microbiota balance, and lead to inflammatory response dysregulation and immune system impairment. Pathological findings have revealed that extended exposure to both antibiotics and disinfectants results in a reduction in colon length, with disinfectants additionally causing damage to the colonic mucosa. Such alterations could disrupt normal gut functions, impairing nutrient absorption and digestion. Disinfectant exposure increases *Staphylococcus* levels, while antibiotics elevate *Bacteroides* concentrations—both types of bacteria lack T3SS, contributing to reduced *NLRC4* activation (77). Disinfectant exposure has also been observed to damage intestinal integrity in mice, further influencing *NLRC4* expression. Consequently, mice subjected to long-term exposure to disinfectants or antibiotics exhibit compromised gut immune functions. Despite distinct alterations in their microbiota, these changes negatively affect their healthy development.

Research has demonstrated that a reduction in *NLRC4* expression can significantly influence subsequent immune pathways. Reduced levels of *NLRC4* may lead to a decline in the production of critical proinflammatory cytokines, including *IL-1β* and *IL-18* (78–81), potentially weakening inflammatory responses and impairing the body’s capacity to eliminate pathogens. Beyond its direct in inflammatory mechanisms, *NLRC4* engages with various immune regulatory pathways, including the activation of the *NLRP3* inflammasome and the stimulation of *caspase-1* and *caspase-11* (82, 83). A diminished expression of *NLRC4* could result in dysregulation of these pathways, adversely affecting cellular responses to infections and pathogen clearance. Within the intestinal environment, *NLRC4* is instrumental in maintaining the equilibrium of the microbial community and preventing the proliferation of pathogens. It regulates inflammatory reactions, safeguarding the integrity of the intestinal mucosal barrier and preventing the intrusion of detrimental microbes (84). A reduction in *NLRC4* expression might lead to dysbiosis of the gut microbiome, increasing the risk for gastrointestinal disorders (84). The chemical constituents of disinfectants may disrupt the equilibrium of gut microbiota and perturb the gut immune environment, notably impacting the expression of critical immune regulatory proteins such as *NLRC4*. The suppression of *NLRC4* expression affects more than just direct pathogen defense; it also alters the expression of subsequent immune mediators, including *IL-18*, *IL-1β*, *NLRP3*, *caspase-1*, and *caspase-11*. These elements are crucial for sustaining intestinal immune responses and managing inflammation. The disturbance of these immune components can precipitate immune system dysfunction, heightening the risk for inflammatory bowel disease and other gut disorders. However, the specific mechanisms underlying these effects remain unclear and require further research.

In conclusion, our findings suggest that while the widespread use of disinfectants is essential for pathogen control, it may inadvertently lead to disorders within the gut microbiome and a weakened immune response. Disinfectants can disrupt the delicate balance of the gut ecosystem, causing an increase in gut *Staphylococcus*, which impairs overall gut function and can trigger a range of immune disorders. Additionally, disinfectants decrease gut *NLRC4* expression. This reduction in *NLRC4*, which is pivotal for producing crucial proinflammatory cytokines and the activation of various immune regulatory pathways, weakens the body’s defenses against pathogens. Furthermore, *NLRC4* plays a crucial role in maintaining the microbial community balance and safeguarding the intestinal mucosal barrier, preventing the overgrowth of harmful microbes and the onset of gastrointestinal diseases such as inflammatory bowel disease. Therefore, this research highlights the critical need for a balanced approach to disinfectant use, advocating for strategies that mitigate potential adverse effects on gut health and the broader immune system.
